# Low-Level Laser Irradiation Improves Functional Recovery and Nerve Regeneration in Sciatic Nerve Crush Rat Injury Model

**DOI:** 10.1371/journal.pone.0103348

**Published:** 2014-08-13

**Authors:** Chau-Zen Wang, Yi-Jen Chen, Yan-Hsiung Wang, Ming-Long Yeh, Mao-Hsiung Huang, Mei-Ling Ho, Jen-I Liang, Chia-Hsin Chen

**Affiliations:** 1 Department of Physiology, Kaohsiung Medical University, Kaohsiung, Taiwan; 2 Orthopaedic Research Center, Kaohsiung Medical University, Kaohsiung, Taiwan; 3 Department of Physical Medicine and Rehabilitation, Kaohsiung Medical University Hospital, Kaohsiung, Taiwan; 4 Department of Physical Medicine and Rehabilitation, Kaohsiung Municipal Ta-Tung Hospital, Kaohsiung Medical University, Kaohsiung, Taiwan; 5 School of Dentistry, College of Dental Medicine, Kaohsiung Medical University, Kaohsiung, Taiwan; 6 Department of Biomedical Engineering, National Cheng Kung University, Tainan, Taiwan; 7 Department of Physical Medicine and Rehabilitation, College of Medicine, Kaohsiung Medical University, Kaohsiung, Taiwan; MGH, MMS, United States of America

## Abstract

The development of noninvasive approaches to facilitate the regeneration of post-traumatic nerve injury is important for clinical rehabilitation. In this study, we investigated the effective dose of noninvasive 808-nm low-level laser therapy (LLLT) on sciatic nerve crush rat injury model. Thirty-six male Sprague Dawley rats were divided into 6 experimental groups: a normal group with or without 808-nm LLLT at 8 J/cm^2^ and a sciatic nerve crush injury group with or without 808-nm LLLT at 3, 8 or 15 J/cm^2^. Rats were given consecutive transcutaneous LLLT at the crush site and sacrificed 20 days after the crush injury. Functional assessments of nerve regeneration were analyzed using the sciatic functional index (SFI) and hindlimb range of motion (ROM). Nerve regeneration was investigated by measuring the myelin sheath thickness of the sciatic nerve using transmission electron microscopy (TEM) and by analyzing the expression of growth-associated protein 43 (GAP43) in sciatic nerve using western blot and immunofluorescence staining. We found that sciatic-injured rats that were irradiated with LLLT at both 3 and 8 J/cm^2^ had significantly improved SFI but that a significant improvement of ROM was only found in rats with LLLT at 8 J/cm^2^. Furthermore, the myelin sheath thickness and GAP43 expression levels were significantly enhanced in sciatic nerve-crushed rats receiving 808-nm LLLT at 3 and 8 J/cm^2^. Taken together, these results suggest that 808-nm LLLT at a low energy density (3 J/cm^2^ and 8 J/cm^2^) is capable of enhancing sciatic nerve regeneration following a crush injury.

## Introduction

Peripheral nerve injury results from various etiologies, such as traction, crushing, ischemic change, cutting injury and long bone fracture, that lead to axonotmesis, in which axons and the covering myelin sheaths are damaged but the connective tissue is preserved, or more severely, neurotmesis, which involves disruption of the entire nerve fiber [1], [2]. Injury to the peripheral nerve results in secondary muscle atrophy, causing various levels of disabilities. Once the peripheral nerve is damaged, degeneration occurs both distal to the injured site through Wallerian degeneration and proximal to the injured site through retrograde degeneration, influencing the corresponding neurons [3]. The sciatic nerve is a large nerve fiber that originates from the lumbosacral plexus with mixed motor and sensory components and is responsible for the motor control and sensory innervation of the lower limbs. Trauma, entrapment and ischemia can cause sciatic nerve damage and lead to limb dysfunction.

Regeneration occurs, albeit slowly, after peripheral nerve injury. Surgical repair is the mainstay for severe or complete nerve injury. Surgical approaches have been developed to repair injured nerves using advanced techniques, such as allograft, autograft and emerging materials science and engineering techniques [4], [5]. Nevertheless, non-surgical approaches have also been developed to facilitate nerve regeneration either for the primary management of axonotmesis or as an adjunctive therapy after surgical repair. The foremost treatment of nerve injury should be rehabilitation programs to maintain adequate joint range of motion and muscle tone to avoid secondary muscle atrophy. Physical therapies such as low frequency electrical stimulation [6]–[8] and magnetic stimulation [9] were proposed to have positive effects on nerve regeneration and functional recovery. Growth factors and neural stem cell transplantation were also claimed to have important neuroprotective effects [10], [11]. However, the high medical expense and invasiveness of these procedures prevents their use in routine clinical practice.

Low-level laser therapy (LLLT) was introduced into medical applications in the 1960s. It is a noninvasive treatment modality that has been applied in various fields, is effective in pain relief and promotes the recovery of some pathologies, including tendinopathies, osteoarthritis, rheumatoid arthritis, wound healing and nerve injuries [12]–[15]. Previous studies have shown positive biological effects of low-level laser stimulation on the nervous system. Several randomized controlled trials applying a low-level laser to an injured peripheral nerve show positive effects with respect to accelerating regenerative processes after the injury [16], [17]. Improved peripheral nerve function leading to significant functional recovery following LLLT was also proposed by Rochkind et al [18].

Animal models of peripheral nerve injury have been developed to evaluate the effect of LLLT in the regeneration of peripheral nerve injury [19]–[21]. Functional, histological, morphological and electrophysiological assessments of the effect of LLLT proved that it had beneficial effects on the regeneration of rat sciatic nerve following an injury [20]–[23]. Shin et al. [24] also found elevated GAP43 immunoreactivity in regenerating peripheral nerves after LLLT, suggesting more rapid neural recovery. Morphologic changes evaluated by light microscopy and electron microscopy were also used to determine the extent of demyelination and vascular changes in the peripheral nerve segment [25], [26]. However, a thorough evaluation of the effects of LLLT using molecular, histological and morphological analyses to assess functional recovery has not been performed, and the optimal parameters of LLLT to facilitate peripheral nerve regeneration are still controversial. The purpose of this study is to determine the effects of LLLT and the effective laser dose to facilitate neural regeneration in a rat sciatic nerve injury model, using both molecular and functional assessments.

## Materials and Methods

### Sciatic nerve crush injury in rat

Sprague Dawley (SD) rats were purchased from the National Laboratory Center (Taipei, Taiwan), and the *in vivo* experiments were performed with the approval of the Kaohsiung Medical University Animal Care and Use Committee. A total of 36 9-week-old SD rats were randomly divided into 6 experimental groups: (1) a normal group without 8 J/cm^2^ LLLT (Normal), (2) a normal group with 8 J/cm^2^ LLLT, in which the animals received no sciatic nerve crush (Normal+8J), (3) a sciatic nerve crush group, in which the animals received a right sciatic nerve crush, without LLLT (Crush), (4) a sciatic nerve crush group with 3 J/cm^2^ LLLT (Crush+3J), (5) a sciatic nerve crush group with 8 J/cm^2^ LLLT (Crush+8J) and (6) a sciatic nerve crush group with 15 J/cm^2^ LLLT (Crush+15J). The food was limited to 15 g per rat every day. To create the sciatic nerve crush injury in the right hindlimb, the rats were anesthetized by intraperitoneal injection of ketamine (Ketalar, Parke-Davis, Taiwan) in combination with xylazine-hydrochloride (Rompun, Bayer HealthCare, Germany). The hair over the posterior hindlimb was shaved and cleaned with a depilator. The skin was sterilized with 10% povidone iodine and then incised 2 cm open over the posterior hindlimb to expose the sciatic nerve. A non-serrated forceps with smoothed surface was used to create a 3-mm-long crush injury at the right sciatic nerve by approximately 54 N for 30 seconds, and the left hindlimb was a sham control surgery without crush injury. The wounds were sutured after surgery with nylon 5-0 sutures. For the LLLT, the rats were treated daily with a total energy of 3, 8 or 15 J/cm^2^ laser irradiation to the center of the surgical site. After twenty days of LLLT, the rats were euthanized using CO_2_ inhalation. The sciatic nerve was quickly removed, embedded in Tissue-Tek OCT compound 4583 (Sakura Finetech, Tokyo, Japan) and frozen in liquid nitrogen. The 5-µm-thick sections of sciatic nerve were cut serially in a cryostat and collected onto glass slides (MAS-GP type A; Matsunami Glass, Osaka, Japan) for immunofluorescence analysis.

### Low-level laser therapy (LLLT)

The Gallium Aluminum Arsenide (Ga-Al-As) near-infrared laser with a wavelength of 808 nm (Transverse IND. CO., LTD., Taipei, Taiwan) was used as the laser source at a laser power output of 170 mW and power density of 44.7 mW/cm^2^. After sciatic nerve crush injury of the rat's right hindlimb, 808-nm LLLT was applied over the sciatic nerve crush site, and a total energy of 3, 8 or 15 J/cm^2^ on the surface of the skin was applied once per day for 20 consecutive days in each intervention group. The laser irradiation parameters are listed in Table 1. The sham-operated sciatic nerve of the rat left hindlimb was subjected to LLLT as indicated.

**Table 1:** The Laser Irradiation Parameters.

Laser type: Ga-Al-As 808 nm wavelength laser					
Laser mode: Continuous wave					
Total energy (J/cm^2^)	Power output(mW)	Spot area(cm^2^)	Power density (mW/cm^2^)	Time irradiation(sec)	Irradiation treatment
3.0	170	3.8	44.7	67.2	Once a day
8.0	170	3.8	44.7	179.0	Once a day
15.0	170	3.8	44.7	335.6	Once a day

### Sciatic functional index (SFI) analysis for functional assessment of sciatic nerve regeneration

The SFI was used as described by De Medinaceli et al [27] to assess the functional recovery of the damaged rat sciatic nerve by comparing the SFI before LLLT and after 20 days of LLLT. The assessment occurred on a transparent acrylic track that was 80 cm long and 6 cm wide with a small, dark shelter at the end. The rats were walked down the track and real-time images were captured via digital camera recording. Then, the footprint images were evaluated using the computer program ImageJ. The SFI was calculated according to the following equation as described by Bain et al [28]:

SFI = −38.3 [(EPL-NPL)/NPL]+109.5 [(ETS-NTS)/NTS]+13.3 [(EITS-NITS)/NITS]−8.8. The print length (PL, the distance from the heel to the third toe), toe spread (TS, distance from the first to the fifth toe) and intermediary toe spread (ITS, distance from the second to the fourth toe) of the experimental (E) and normal (N) sides were measured from the recorded footprints in a gait track.

### Range of motion (ROM) analysis for functional assessment of sciatic nerve regeneration

Functional assessments of nerve regeneration by ROM were measured to evaluate the degree of functional recovery before and after LLLT. After 20 days, the ROM of the experimental (right hindlimb) and control (left hindlimb) hind feet was measured during the mid-stance phase of the gait cycle.

### Transmission electron microscopy (TEM) analysis for evaluation of the myelin sheath thickness in sciatic nerve

The TEM imaging grids were prepared by dropping centrifuged solutions onto a carbon-coated copper grid followed by drying. TEM images of the sciatic nerve were captured and conducted using a JEM-2000EXII transmission electron microscope (Japan Electron Optics Laboratory Co., Ltd., Tokyo, Japan) operating at 200 kV. An energy dispersive spectroscopy system (Link II Energy Dispersive X-ray Analysis System, England) attached to the microscope was used to determine the chemical characterization of the sample. The myelin sheath thickness in the TEM images was measured using an image analysis system (Image-Pro Plus; Media Cybernetics Inc., Silver Spring, MD, USA).

### Western blotting analysis for detecting the GAP43 expression in sciatic nerve

A sciatic nerve segment with its proximal 1 cm and distal 1 cm lengths was obtained. The sciatic nerve segment was washed twice with ice-cold PBS containing 1 mM sodium vanadate and lysed in a modified radio immunoprecipitation assay buffer (RIPA: 150 mM NaCl, 1 mM EGTA, 50 mM Tris [pH 7.4], 10% glycerol, 1% Triton X-100, 1% sodium deoxycholate and 0.1% SDS) with protease inhibitors (Complete Protease Inhibitor Cocktail Tablets; Roche Diagnostics Ltd., Taipei, Taiwan) and 1 mM sodium vanadate. The lysates were cleared using centrifugation at 14,000 rpm for 15 min at 4°C and analyzed using western blotting with anti-GAP43 (Abcam, Cambridge, MA) and anti-β-actin (Sigma-Aldrich, St. Louis, MO) antibodies. The membranes were developed using the Immobilon Western HRP Substrate (Millipore, Billerica, MA). The blots were digitally evaluated and measured using the UVP AutoChemi™ Image and Analysis System (UVP, Upland, CA).

### Immunofluorescence for detecting GAP43 and neurofilament in sciatic nerve

The 5-µm-thick sections of sciatic nerve on glass slides were washed three times with PBS and then fixed with 4% paraformaldehyde in PBS for 20 min at room temperature. After washing three times with PBS, the cells were permeabilized with 0.5% Triton X-100 in PBS for 10 min, rinsed with PBS and then immunostained with anti-GAP43 (Abcam) and neurofilament-M (Chemicon) antibodies for 1 h at room temperature. After washing three times with PBS, the glass slides were exposed to AlexaFluor 488-labeled and AlexaFluor 596-labeled secondary antibodies (Molecular Probes, Inc., Eugene, OR) for 1 h, and then the nuclei were stained with DAPI. The coverslips were mounted in anti-fade solution (Molecular Probes). Images of the samples were captured on a fluorescence microscope. In each specimen, staining without the primary antibody was performed in a side-by-side parallel specimen as a negative control, and all controls yielded a blank image.

### Statistical analysis

A total of 36 SD rats were divided into 6 groups and there were 6 rats per group. All data were expressed as the mean±SD. Statistical significance was evaluated by one-way analysis of variance (ANOVA), and multiple comparisons were performed using Scheffe's method. A P value of less than 0.05 was considered statistically significant.

## Results

### LLLT at both 3 and 8 J/cm^2^ improved SFI in the sciatic nerve-crushed rats

**Figure 1:**
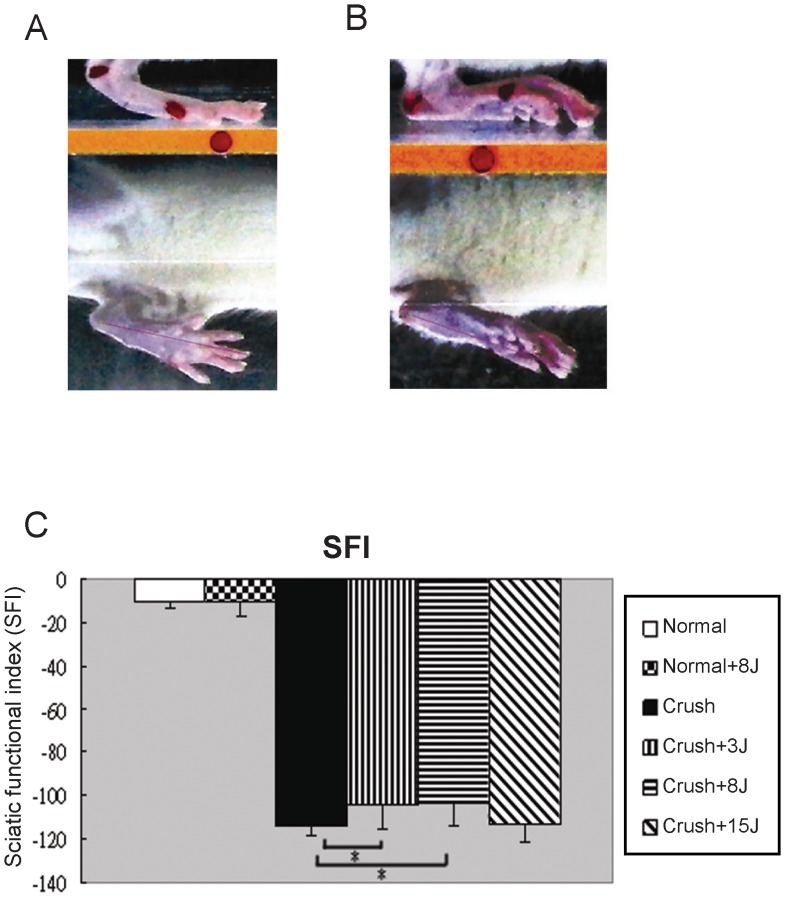
The 3 J/cm^2^ and 8 J/cm^2^ LLLT treatments improved the sciatic functional index (SFI) in rats with a sciatic nerve crush injury. Representative images of (A) the sham-operated rat left hindlimb and (B) the sciatic-injured rat right hindlimb. (C) Functional assessment of the recovery of injured sciatic nerves by calculating the SFI in the normal rats without (normal) or with 8 J/cm^2^ LLLT (normal+8J) and in the sciatic nerve-crushed rats without (crush) or with LLLT at 3 J/cm^2^ (crush+3J), 8 J/cm^2^ (crush+8J) or 15 J/cm^2^ (crush+15J). *P<0.05, compared with crush group.

To examine the functional recovery in rats with a sciatic nerve crush, we detected the SFI and ROM using gait analysis. The sham-operated rat left hindlimb was able to raise the heel and open the toes (Fig. 1A), and the SFI was approximately −10%. After the crush injury to the rat's sciatic nerve, the hindlimb was unable to raise the heel and open the toes (Fig. 1B), and the SFI decreased to −120% (Fig. 1C). These results demonstrated effective sciatic nerve injury leading to declined locomotion. After 20 days, we found significant improvement of the SFI in the groups receiving 3 and 8 J/cm^2^ LLLT compared with the crush group without irradiation. However, we did not observe significant SFI improvement in the 15 J/cm^2^ LLLT group (Fig. 1C).

### LLLT at 8 J/cm^2^ improved ROM in the sciatic nerve-crushed rats

**Figure 2:**
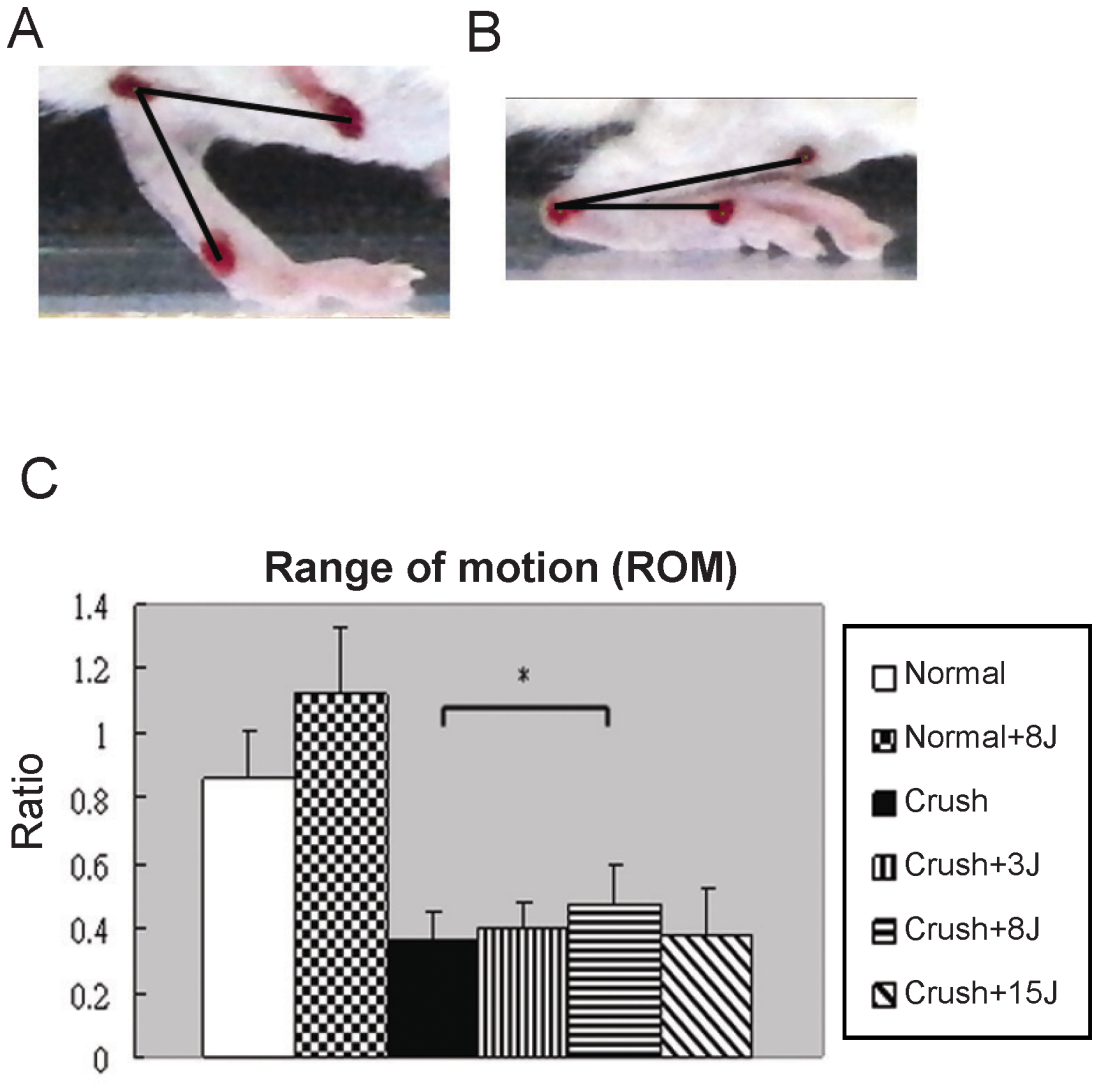
The 8 J/cm^2^ dosage of 808-nm laser enhanced the range of motion (ROM). (A) Representative images show the sham-operated left rat hindlimb at mid-stance phase and (B) the sciatic-injured right rat hindlimb at mid-stance phase. (C) Functional assessment of the recovery of the injured sciatic nerve using ROM analysis by comparing the right hindlimb with the left hindlimb (ratio) in the normal rats without (normal) or with 8 J/cm^2^ LLLT (normal+8J) and in the sciatic nerve-crushed rats without (crush) or with LLLT at 3 J/cm^2^ (crush+3J), 8 J/cm^2^ (crush+8J) or 15 J/cm^2^ (crush+15J). *P<0.05, compared with the crush group.

Fig. 2A shows that the sham-operated left rat hindlimb had a large ROM angle when the foot was at the mid-stance phase whereas the sciatic-injured right rat hindlimb had a small ROM angle when the foot was at the mid-stance phase (Fig. 2B). The ROM of each hindlimb was measured during the mid-stance phase on both sides, and the ratio of the injured (right hindlimb) to the intact (right hindlimb) side was calculated (Fig. 2C). As shown in Fig. 2C, a significant improvement in the ROM was found in rats receiving 8 J/cm^2^ LLLT after the sciatic nerve crush injury. With lower (3 J/cm^2^) or higher (15 J/cm^2^) doses, no further improvement was found.

### LLLT enhanced the myelin sheath thickness in the sciatic nerve-crushed rats

**Figure 3:**
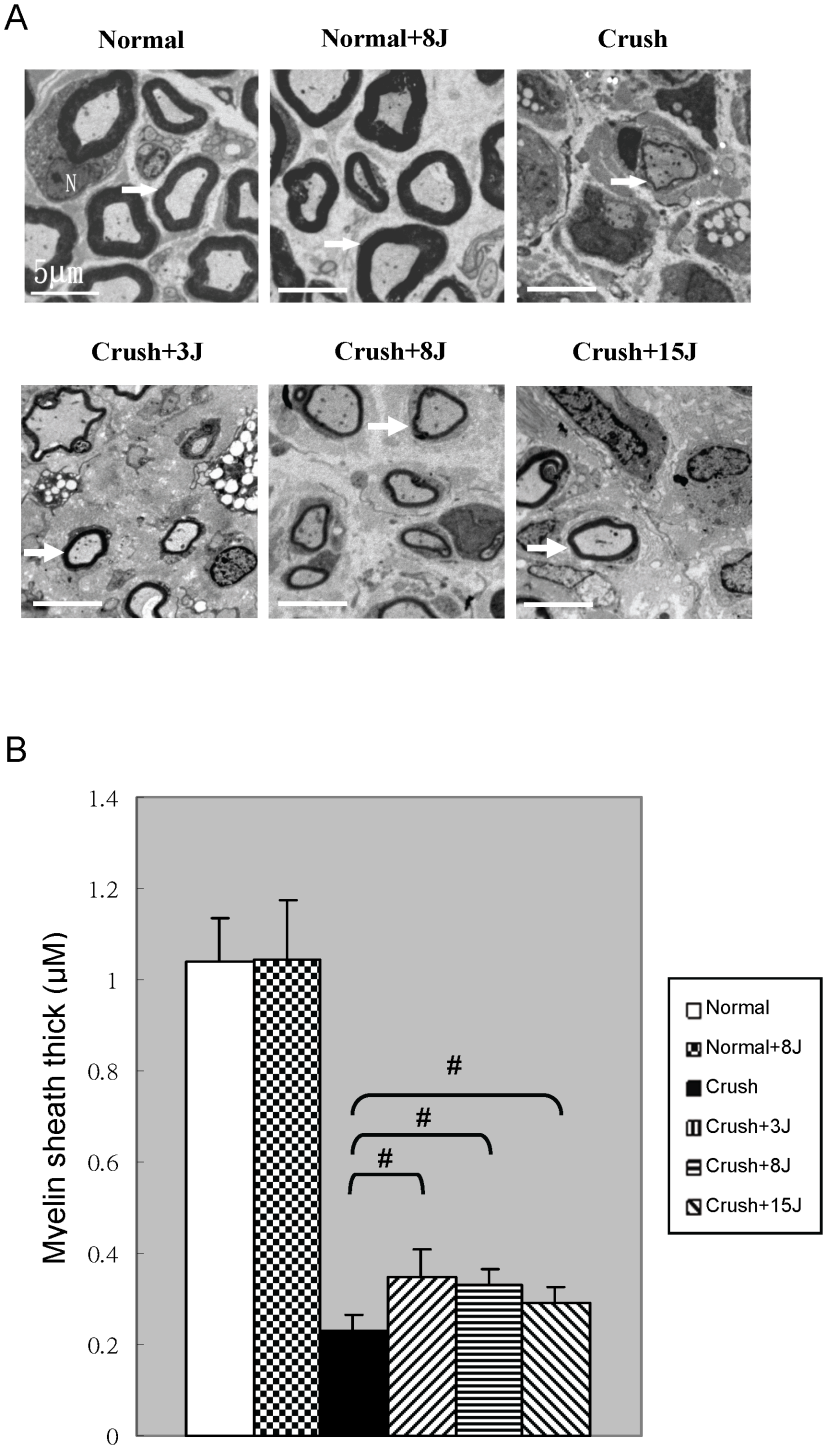
LLLT-treated (808 nm) sciatic nerve presented thicker myelin sheaths. Representative TEM images (A) and quantitative analyses of myelin sheath thickness (B) in the sciatic nerves of sham-operated rats without (normal) or with 8 J/cm^2^ LLLT (normal+8J) and in the rats with sciatic nerve crush injury without (crush) or with LLLT at 3 J/cm^2^ (crush+3J), 8 J/cm^2^ (crush+8J) or 15 J/cm^2^ (crush+15J). ^#^P<0.05, compared with the crush group. Scale bar, 5 um.

Regeneration after axonotmesis occurs with reactive Schwann cells and preserved endoneurium. We investigated the sciatic nerve regeneration using TEM analysis to evaluate the myelin sheath thickness. As shown in Fig. 3A, the TEM micrographs revealed a dense myelin sheath around the normal sciatic nerve. The integrity of the myelin sheath was obviously disrupted after the crush injury. The myelin sheath thickness analysis showed that the sciatic nerve of the normal group had a thicker myelin sheath than that of the crush group. In the sciatic nerve-crushed groups, the groups treated with LLLT presented significantly thicker myelin sheaths than the untreated crush group (Fig. 3B).

### LLLT enhanced GAP43 expression in the sciatic nerve-crushed rats

**Figure 4:**
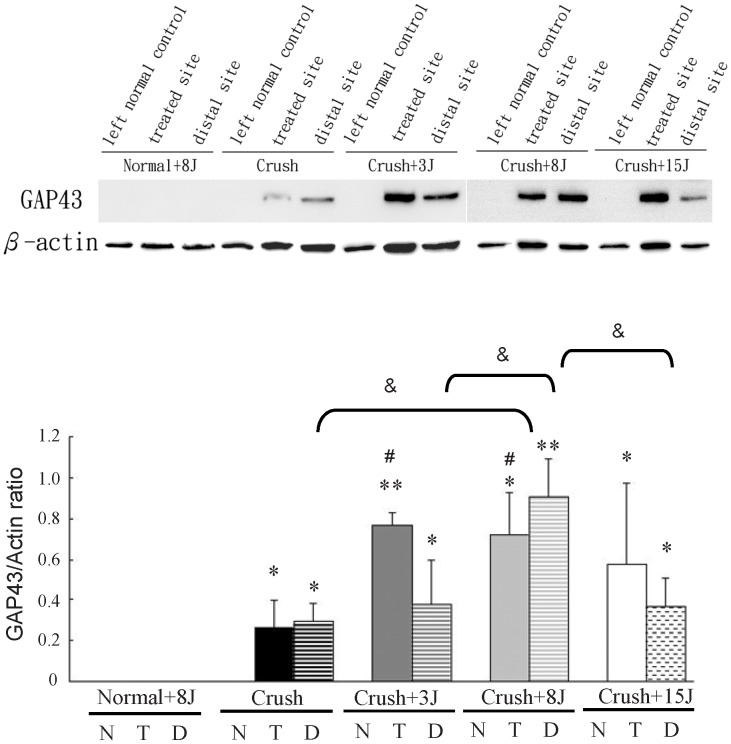
Detection of GAP43 protein expression in rats with sciatic nerve crush injury after 808-nm LLLT treatments using western blot analysis. Representative western blotting and quantitative analyses of GAP43 protein expression in the sciatic nerve of normal rats with 8 J/cm^2^ LLLT (normal+8J) and in the rats with sciatic nerve crush injury without (crush) or with LLLT at 3 J/cm^2^ (crush+3J), 8 J/cm^2^ (crush+8J) or 15 J/cm^2^ (crush+15J) were shown. We detected the expression of the GAP43 protein in the sciatic nerve of left sham-operated hindlimb (left normal control; N) and in the sciatic nerve of right hindlimb at the laser-treated site (T) or a distal site (D). *P<0.05, **P<0.01, compared with the left sham-operated hindlimb (left normal control; N). ^#^P<0.05, compared with the crush group at the laser-treated site (T). ^&^P<0.05, compared between the crush groups at the distal site (D).

**Figure 5:**
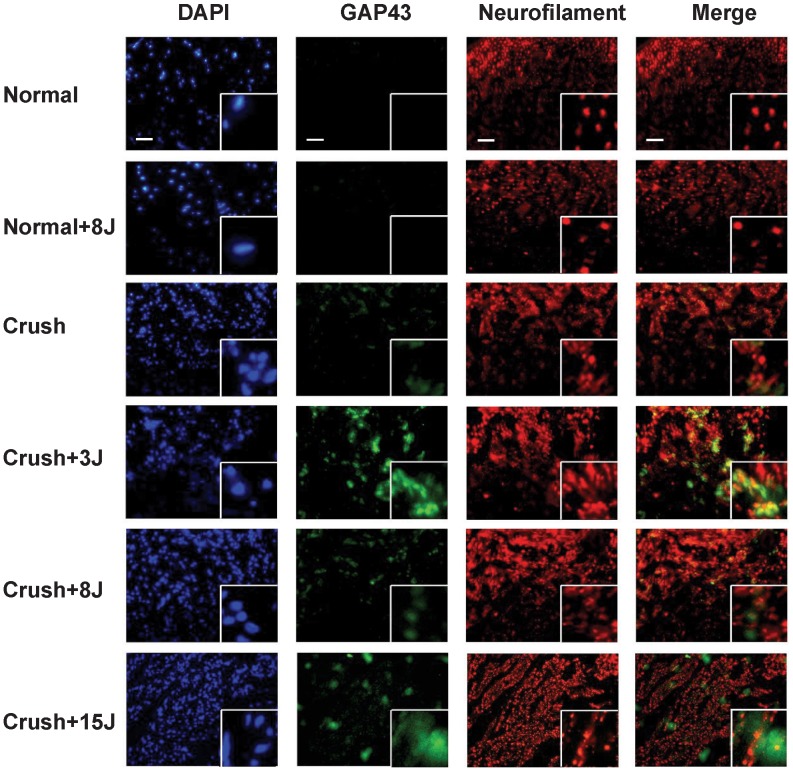
Detection of GAP43 expression in the sciatic nerve of 808-nm LLLT laser-treated site using immunofluorescent staining. Groups: sham-operated rats without (normal) or with 8 J/cm^2^ LLLT (normal+8J) and rats with sciatic nerve crush injury without (crush) or with LLLT at 3 J/cm^2^ (crush+3J), 8 J/cm^2^ (crush+8J) or 15 J/cm^2^ (crush+15J). Sections were labeled with DAPI (blue), GAP43 (green) and neurofilament (red), which is specifically expressed in neurites. Original magnification: 100×. White boxes show the enlarged views with a magnification of 400×. Scale bar, 200 um.

**Figure 6:**
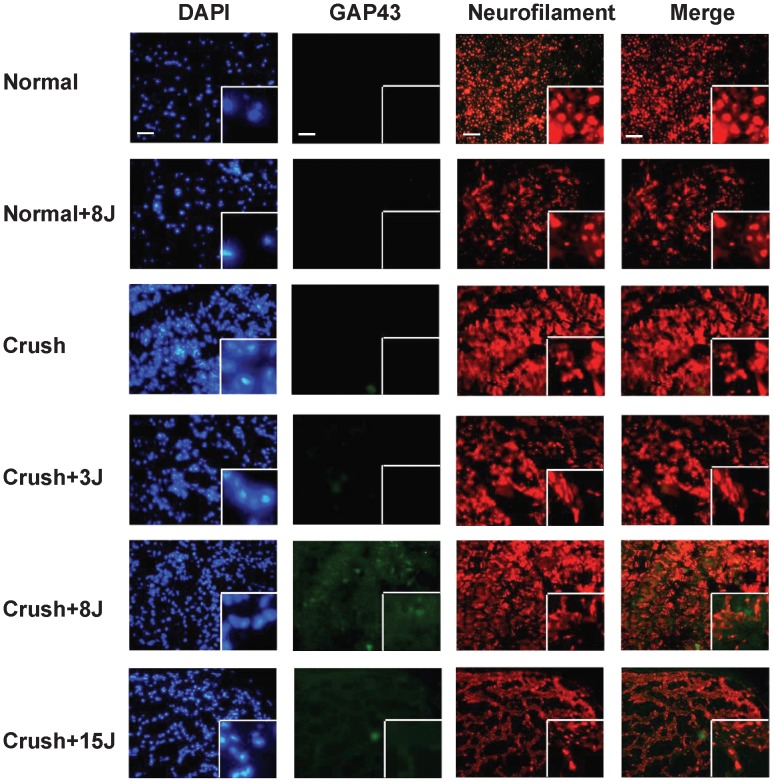
Detection of GAP43 expression in the sciatic nerve of 808-nm LLLT laser-treated distal site using immunofluorescent staining. Groups: sham-operated rats without (normal) or with 8 J/cm^2^ LLLT (normal+8J) and rats with sciatic nerve crush injury without (crush) or with LLLT at 3 J/cm^2^ (crush+3J), 8 J/cm^2^ (crush+8J) or 15 J/cm^2^ (crush+15J). Sections were labeled with DAPI (blue), GAP43 (green) and neurofilament (red), which is specifically expressed in neurites. Original magnification: 100×. White boxes show the enlarged views with a magnification of 400×. Scale bar, 200 um.

In the sciatic nerve-crushed rats that received 3, 8 and 15 J/cm^2^ LLLT, the expression of the regeneration marker GAP43 was determined using western blotting (Fig. 4) and immunofluorescence analysis (Fig. 5 and Fig. 6) at the laser-treated site and a distal site of the sciatic nerve. The results showed that in normal rats with 8 J/cm^2^ LLLT, the GAP43 protein was not expressed at the sciatic nerve of the left normal hindlimb (normal) or the sciatic nerve of right hindlimb at the laser-treated (Fig. 4 and Fig. 5) or distal site (Fig. 4 and Fig. 6). In the sciatic nerve-crushed groups, the GAP43 protein levels were significantly higher after LLLT compared with groups without laser irradiation. In the laser-treated site, the 3 J/cm^2^-treated group showed higher levels of GAP43 expression than the 8 J/cm^2^-treated group (Fig. 4 and Fig. 5), whereas there was no additional effect on the left normal sciatic nerve control group (Fig. 4). We also found that in the nerve distal to the crush site, the 8 J/cm^2^-treated group showed even higher GAP43 expression levels than the 3 J/cm^2^ and 15 J/cm^2^ groups (Fig. 4 and Fig. 6).

## Discussion

The present study evaluated the effects of low-level laser irradiation on peripheral nerve regeneration following rat sciatic nerve crush injury. The results indicated that the application of 808-nm LLLT at 3 and 8 J/cm^2^ for 20 days is effective in the promotion of sciatic nerve regeneration, as determined by molecular, morphologic and functional assessments.

Peripheral nerve injuries lead to Wallerian degeneration distal to the lesion, involving axonal loss and myelin sheath degradation, and retrograde degeneration proximal to the lesion, involving nerve cell body degradation [3]. The sciatic nerve is responsible for the motor control and sensory innervation of the lower limbs. Injury to the sciatic nerve could result in secondary muscular atrophy, resulting in varying degrees of disabilities. Although self-repair of the peripheral nerve is possible, the regeneration is slow. Microsurgical approaches have been advanced [4], [5]; nevertheless, non-surgical approaches such as physical modalities have also been proposed to facilitate nerve regeneration [6], [8], [9].

LLLT has been widely applied in clinical practice for the facilitation of nerve regeneration. Studies have shown that Schwann cells, the principal glial cells of the peripheral nervous system, secrete neurotrophic factors that promote the regeneration of the peripheral nerve [29], [30]. Phototherapy can stimulate Schwann cell proliferation in vitro [31]. In vivo studies [20]–[23], [32] assessing functional recovery, histological and micro-morphological changes and electrophysiological improvement after the introduction of LLLT proved to have beneficial effects on the regeneration of rat sciatic nerve injury. LLLT also accelerated the repair of transected sciatic nerves in addition to nerve conduits for gapped peripheral nerves [32], [33]. Moges et al. reported that light therapy for 14 consecutive days using 810 nm LLLT at energy density of 25 J/cm^2^ enhanced nerve regeneration and accelerated functional recovery after autologous nerve graft repair using fibrin glue in a rat model of median nerve transection injury [32]. The irradiation parameters described were different from our present study, in which we showed that light therapy for 20 consecutive days using 808-nm LLLT at a low energy density (3 J/cm^2^ and 8 J/cm^2^) accelerated functional recovery and enhanced nerve regeneration in sciatic nerve crush rat injury model.

Although various assessments are used with regard to peripheral nerve regeneration, functional recovery is thought to be the primary consideration that links most directly to improved functional performance after peripheral nerve injury. The SFI proposed by Bain et al is one of the most commonly used parameters to assess the functional recovery of damaged rat sciatic nerves [28]. In the study of Shen et al, correlating SFI to muscle strength, electrophysiological and morphological assessments after peripheral nerve injury or repair, indicated that the SFI is a reliable index for evaluating rat sciatic nerve regeneration [34]. However, the reliability of the SFI should be carefully assessed when using it as a single parameter to evaluate more severe sciatic nerve injuries, such as transected sciatic nerve with gaps or longer segment of crush injury. Shenaq et al assessed the correlation of SFI to histological results and clinical observations in rat sciatic nerve regeneration with a 1-cm gap and claimed no definite correlation in all groups over various periods postoperatively [35]. Another study by Monte-Raso et al evaluated the reproducibility of the SFI in rats with severe crush injury using a 5000-g static load on a 5-mm segment of the sciatic nerve [36]. They concluded that the SFI parameter is only reliable beginning at three weeks after a severe lesion of the sciatic nerve. In studies of Varejão et al [37] and Luís et al [38], a non-serrated clamp exerting a force of 54 N was used to create a sciatic nerve crush injury. In addition to functional assessment of reinnervation with the SFI, extensor postural thrust (EPT), withdrawal reflex latency (WRL) and kinematic analysis, morphological assessments such as myelin thickness and axon diameter were also evaluated. They recommended the combined use of various methods for a more thorough assessment of sciatic nerve regeneration and functional recovery.

Another functional evaluation is the clinical observation of gait patterns and the measurement of ankle kinematics. The ankle angle is representative of plantarflexion and dorsiflexion during the stance phase and is an important parameter in assessing the gait pattern [39]. Lin et al developed a motor functional index, the ankle stance angle, to assess rat sciatic nerve regeneration, which revealed a significant correlation with ankle joint passive ROM, and suggested an adequate reliability in the evaluation of functional recovery [40]. Computerized gait analysis also emerged in the application of assessing the recovery of hindlimb locomotion [37], [41]. In our study, we calculated the ratio of injured to non-injured ankle joint ROM and observed a significant decrease in ankle joint ROM ratio after crush injury. We also observed a significant improvement of ankle joint ROM ratio after 8 J/cm^2^ LLLT, which was compatible with previous study results claiming the effective use of LLLT in the promotion of sciatic nerve regeneration.

The present study assessed morphological changes by TEM and observed thicker myelin sheaths with more dense alignments in the 3 and 8 J/cm^2^ irradiated groups. A previous study by Mohammed et al [42] applied low-level laser irradiation to New Zealand adult rabbits with complete transection of the peroneal nerve and observed significant structural changes, such as thicker nerve fibers, more regular myelin layers and clearer nodes of Ranvier with the absence of a short node after laser treatment. Bae et al assessed the morphological changes of rat sciatic nerves after low-level laser irradiation using both light and electron microscopy and reported increased numbers of myelinated axons and decreased numbers of degenerated axons in the irradiated group compared with the control group without irradiation, which were proportional to the length of the laser treatment [22]. In addition to changes in myelination, increased numbers of Schwann cells were also found after LLLT [23], [31], suggesting the promotion of peripheral nerve regeneration through the secretion of various neurotrophic factors by Schwann cells.

GAP43 is expressed at high levels in nerve growth cones during development and is present in regenerating peripheral nerves [43], [44]. Shin et al found elevated GAP43 immunoreactivity in regenerating peripheral nerves after LLLT. The elevated GAP43 immunoreactivity peaked at 3 weeks after injury in the irradiated group and declined at 5 weeks in both the irradiated and non-irradiated groups, with no significant differences between the 2 groups. These findings emphasize the effect of LLLT on the early stages of the nerve recovery following sciatic nerve injury [24]. Thus, LLLT should be introduced immediately after the nerve injury. Our present study applied 20 consecutive days of low level laser irradiation immediately after a rat sciatic nerve crush injury, and the results showed increased levels of GAP43 expression in the laser-treated groups. The results were consistent with previous studies. We also analyzed the GAP43 expression level at 1 cm distal to the injured sciatic nerve, which suggested significantly higher levels of expression in the group treated with 8 J/cm^2^ laser irradiation. LLLT at 8 J/cm^2^ may have had a stronger influence on the injured sciatic nerve tissue by promoting increased regeneration efficiency and extending GAP43 expression to sites distal to the crushed region.

Varying effects of LLLT have been reported; negative effects of laser therapy have been reported by Chen et al [45]. A 904-nm GaAs laser source with pulsed laser irradiation was used over the rat sciatic nerve with a 10-mm gap, beginning a week after nerve repair. The results showed a significantly lower percentage of successful regeneration, a less mature structural organization with a smaller cross-sectional area and a lower number of myelinated axons in the laser-treated groups compared with the control group. Bagis et al also claimed no effect of a GaAs laser (904 nm) on the intact skin of the injured rat sciatic nerve, based on a lack of significant differences in the action potential parameters and histological assessments [46]. Carla et al demonstrated that no differences in recovery were observed in animals with sciatic nerve injury receiving 808-nm LLLT at 10 or 50 J/cm^2^ [47]. The irradiation parameters described were different from our present study, in which we showed that an 808-nm GaAlAs near-infrared laser applied with continuous irradiation to a fluence of 3 J/cm^2^ and 8 J/cm^2^ to an injured rat sciatic nerve immediately after the crush injury was able to provide functional gait recovery and led to increases in myelin sheath thickness and the neuronal growth marker GAP43. The most effective laser application, with regard to optimal wavelength, energy density and pulsed or continuous wave, is still controversial, but an early introduction of LLLT is suggested to facilitate nerve regeneration [24].

In the present study, we determined the immediate effect of LLLT after sciatic nerve injury using 20 consecutive days of irradiation on the functional recovery and nerve regeneration in sciatic nerve crush rat. The optimal treatment duration and long-term effects of LLLT will be evaluated in the future. Further investigations on the differences in proteomic expression in sciatic nerve after receiving LLLT will be conduct to clarify the signaling pathways of the biological effects, including controlling activation of the GAP43 gene, induced by LLLT.

## Conclusion

Based on the results of the present study, we demonstrate that the application of a laser source with an 808-nm wavelength at doses of 3 and 8 J/cm^2^ to an injured rat sciatic nerve immediately after crush has beneficial effects on sciatic nerve regeneration, including better functional recovery and morphological changes and the increased expression of the neuronal growth marker GAP43.
